# Linkage study of fibrinogen levels: the Strong Heart Family Study

**DOI:** 10.1186/1471-2350-9-77

**Published:** 2008-08-12

**Authors:** Lyle G Best, Kari E North, Xia Li, Vittorio Palmieri, Jason G Umans, Jean MacCluer, Sandy Laston, Karin Haack, Harald Goring, Vincent P Diego, Laura Almasy, Elisa T Lee, Russell P Tracy, Shelley Cole

**Affiliations:** 1 Missouri Breaks Industries Research Inc, Timber Lake, SD, USA; 2 University of North Carolina, Chapel Hill, NC, USA; 3 Weill Medical College of Cornell University, New York, NY, USA; 4 Medstar Research Institute, Washington, DC, USA; 5 Southwest Foundation for Biomedical Research, San Antonio, TX, USA; 6 University of Oklahoma Health Sciences Center, Oklahoma City, OK, USA; 7 Laboratory for Clinical Biochemistry Research, University of Vermont, Burlington, Vermont, USA

## Abstract

**Background:**

The pathogenesis of atherosclerosis involves both hemostatic and inflammatory mechanisms. Fibrinogen is associated with both risk of thrombosis and inflammation. A recent meta-analysis showed that risk of coronary heart disease may increase 1.8 fold for 1 g/L of increased fibrinogen, independent of traditional risk factors. It is known that fibrinogen levels may be influenced by demographic, environmental and genetic factors. Epidemiologic and candidate gene studies are available; but few genome-wide linkage studies have been conducted, particularly in minority populations. The Strong Heart Study has demonstrated an increased incidence of cardiovascular disease in the American Indian population, and therefore represents an important source for genetic-epidemiological investigations.

**Methods:**

The Strong Heart Family Study enrolled over 3,600 American Indian participants in large, multi-generational families, ascertained from an ongoing population-based study in the same communities. Fibrinogen was determined using standard technique in a central laboratory and extensive additional phenotypic measures were obtained. Participants were genotyped for 382 short tandem repeat markers distributed throughout the genome; and results were analyzed using a variance decomposition method, as implemented in the SOLAR 2.0 program.

**Results:**

Data from 3535 participants were included and after step-wise, linear regression analysis, two models were selected for investigation. Basic demographic adjustments constituted model 1, while model 2 considered waist circumference, diabetes mellitus and postmenopausal status as additional covariates. Five LOD scores between 1.82 and 3.02 were identified, with the maximally adjusted model showing the highest score on chromosome 7 at 28 cM. Genes for two key components of the inflammatory response, i.e. interleukin-6 and "signal transducer and activator of transcription 3" (*STAT3*), were identified within 2 and 8 Mb of this 1 LOD drop interval respectively. A LOD score of 1.82 on chromosome 17 between 68 and 93 cM is supported by reports from two other populations with LOD scores of 1.4 and 1.95.

**Conclusion:**

In a minority population with a high prevalence of cardiovascular disease, strong evidence for a novel genetic determinant of fibrinogen levels is found on chromosome 7 at 28 cM. Four other loci, some of which have been suggested by previous studies, were also identified.

## Background

Hypotheses concerning the pathogenesis of atherosclerosis have frequently included both hemostatic and inflammatory mechanisms. Fibrinogen levels are positively associated with risk of thrombosis [1] and are also an indicator of acute inflammatory response [2,3]. A recent, large meta-analysis [4] concluded that for each 1 g/L increase in plasma fibrinogen, individuals increase their relative risk of coronary heart disease (CHD) by 1.8 after adjustment for major cardiovascular disease (CVD) risk factors. The Strong Heart Study (SHS) is an ongoing, longitudinal cohort study of CVD among American Indians in three regions of the US, and began in 1988. The current report is derived from the Strong Heart Family Study (SHFS), an extension of the SHS, in which we have previously corroborated the ability of fibrinogen to predict CVD events [5]. The above studies have identified fibrinogen as a predictor of CVD independent of other standard risk factors [6,7], however it is possible that fibrinogen levels are simply correlated with other causal inflammatory factors [8].

Fibrinogen levels are increased by many non-genetic factors, e.g. advancing age, smoking, obesity, oral contraceptive use and estrogen replacement therapy, whereas moderate alcohol intake has been related to lower fibrinogen levels [7,9,10]. Acquired metabolic conditions, e.g. obesity, insulin resistance and type 2 diabetes also increase fibrinogen levels [7,11]. Correlation between fibrinogen and other hemostatic/inflammatory markers has been demonstrated [12,13].

Moderate heritabilities of 34% and 37% were found in two primarily Caucasian populations [9,14]. In our previous work, we demonstrated a heritability between 33 ± 6% and 44 ± 6% among the American Indian participants of the SHFS [15]. Genome-wide linkage studies of fibrinogen [16-18] are uncommon; but have identified a number of suggestive quantitative trait loci (QTL). Polymorphisms of the fibrinogen gene have been extensively investigated for their influence on circulating protein levels [19-24].

Cardiovascular disease accounts for a large proportion of mortality and morbidity in American Indian (AI) communities [25]. Since a substantial number of individuals with CVD events have no previously identifiable risk factors [6], it is important to develop the means to more accurately predict CVD events. Although fibrinogen has been shown to provide risk information independent of HDL, total cholesterol and other typical cardiovascular risk factors [4,6]; a better understanding of the genetic and environmental factors that influence variation in circulating fibrinogen levels may improve our understanding of CVD.

## Methods

The Strong Heart Study (SHS) is a population-based, cohort study of cardiovascular disease among American Indians. The participating communities, study design, survey methods and laboratory techniques have been described previously [26,27]. In 1998, the Strong Heart Family Study, was initiated and participants 16 years and older were recruited, without regard to disease status, from multi-generational families, including index members of the SHS cohort. All participants have given informed consent for the genetic study of CVD and associated risk factors, including the present study. In addition, approval for this study was obtained from relevant tribal communities and institutional review boards.

The protocols for collection of all phenotypic data were briefly described in previous publications [26]. Fibrinogen was measured with a coefficient of variation (CV) of < 8%, using the Clauss method [28] incorporating STA Fibrinogen-5 reagents on the STA-R platform, both from Diagnostica Stago. "Ever" smoking was defined as having smoked at least 100 cigarettes during the lifetime and "current" smoking (present, regular use of smoke tobacco). "Current" and "ever" consumption of alcohol was defined as having had at least 12 alcoholic beverages in the last year or in past years, respectively.

The procedures for genotyping in the SHFS have been described previously [29]. In brief, DNA was isolated from fasting blood samples using organic solvents, and then amplified in separate PCR reactions with primers specific for short tandem repeat markers using the ABI PRISM Linkage Mapping Set-MD10 Version 2.5 (Applied Biosystems, Foster City, CA). PCR products were loaded into an ABI PRISM 377 DNA sequencer for laser-based automated genotyping. Analyses and assignment of the marker alleles were done using computerized algorithms (Applied Biosystems). deCODE Genetics provided sex-averaged chromosomal maps (in units of Haldane centimorgans) for this analysis. Pedigrees were screened with the PREST (Pedigree Relationship Statistical Tests) [30] and Sim Walk2 [31] programs for mendelian inconsistencies and possible double recombinants. The above screening resulted in less than 1% of all genotypes being excluded.

### Statistical analysis

SAS, *version 8.0*, was used to screen covariates for statistical significance using stepwise linear regression by center. Univariate quantitative genetic analyses were used to partition the phenotypic variance into its' additive genetic and environmental components, using maximum likelihood variance decomposition methods [32]. This approach was implemented in the computer program SOLAR, version 2.0 [32] and allows for an explicit test of whether correlations among family members are in part due to genetic effects.

The use of the variance component approach requires an estimate of the identity-by-descent (IBD) matrix. We used the Loki package [33], which employs a Markov chain Monte Carlo stochastic procedure to compute the IBD allele sharing at points throughout the genome conditional on the genotype information available at neighboring points.

A total of 3535 SHFS participants were considered for analysis (Arizona (AZ) = 1195, Dakota (DA) = 1158, Oklahoma (OK) = 1182) after excluding those individuals with missing covariate data and as indicated below to normalize the phenotypic trait distribution. Because variance components methods are sensitive to kurtosis [34], all phenotypic outliers (here defined as any value more than 3 standard deviations from the mean) were removed prior to analysis (number excluded varies by analysis, N < 15). In addition, fibrinogen levels were natural log transformed. All analyses were conducted separately for each center and then on the combined data from all three centers. To maximize our power to detect genetic effects, a minimally adjusted model (Model 1), incorporating age, sex, age by sex and center covariates was analyzed first. Secondary analyses considered adjustment for the linear fixed effects of the covariates listed in Table 1, all of which were identified from the published literature. Covariates whose effects were significant (P < 0.05) in the initial analysis in at least one Center were retained in the maximally adjusted model of all Centers (Model 2), even if the significance levels decreased after inclusion of other covariates. This process resulted in the addition of waist circumference, the presence of ADA defined diabetes [35] and postmenopausal status to Model 2. The addition of current smoking as a covariate resulted in variable and insignificant changes to the outcome, compared with Model 2. We additionally confirmed the significance of Model 2 covariates while accounting for family relationships in SOLAR. Residuals were generated for both models (Model 1 and Model 2) and used in all subsequent genetic analyses. Kurtosis values for fibrinogen were < 0.50 for all analyses.

**Table 1:** Descriptive characteristics of SHFS participants stratified by study recruitment center.^1^

	AZ	DK	OK
Total sample size ^2^	N = 1195	N = 1158	N = 1182
Female N, (%)	747 (63)	684 (59)	700 (59)
Age, years Mean (± SD)	36.99 (16)	38.71 (17)	43.49 (17)
Fibrinogen, mg/dl Mean (± SD)	417.5 (87)	366.2 (80)	384.3 (84)
Diabetes ^3 ^N (%)	391 (33)	162 (14)	241 (20)
Smoking,^4^Current N (%)	301 (25)	498 (43)	391 (33)
Waist, cm Mean (± SD)	111.5 (19)	99.4 (17)	101.6 (16)
Menopausal, Yes N (%)	187 (25)	180 (26)	259 (37)
Alcohol, Current N (%)	704 (59)	772 (67)	563 (48)
Total Cholest, mg/dl Mean (± SD)	174.5 (34.1)	181.4 (36.8)	185.9 (37.2)
LDL-Cholest, mg/dl Mean (± SD)	93.9 (25.9)	100.6 (31.0)	100.1 (30.4)
Triglycerides, mg/dl Mean (± SD)	170.1 (134.6)	156.9 (135.9)	172.3 (171.7)
Estrogen use, Yes N (%)	30 (4)	58 (8)	112 (16)
BMI, Kg/m^2 ^Mean (± SD)	35.3 (8.4)	30.1 (6.7)	31.1 (6.7)

^1 ^Percentages and means calculated only from those with available measurements. ^2 ^Participants remaining after excluding age, waist and fibrinogen ± 3SD and those with missing values. ^3 ^Diabetes was determined using the American Diabetes Association criteria. ^4 ^Smoking was defined as having had at least one hundred cigarettes.

The power of this study for each center was estimated by simulations, assuming fully informative markers. The simulations were based on a total heritability of the fibrinogen phenotype either 25% or 50%. An estimate of the power for all centers combined was determined by calculations based on the results from the individual centers, due to the otherwise extremely high computational demands for simulations.

## Results

The statistical descriptors of fibrinogen and other covariates are stratified by center and displayed in table 1. The unadjusted fibrinogen levels were highest (417.5 mg/dl ± 87) in the Arizona center and the lowest (366.7 mg/dl ± 80) in the Dakota center. Individuals from the Arizona Center had the highest prevalence of diabetes and obesity. In contrast, SHFS participants from the Dakotas Center had the highest prevalence of current smokers. The highest prevalence of menopause was in the Oklahoma Center, perhaps since they were older on average.

Table 2 presents all suggestive (LOD ≥ 1.8) [36] genome-wide, multipoint linkage scores of fibrinogen by center and model. A QTL for fibrinogen (LOD = 3.02) was detected in the Dakota Center on chromosome 7 at 28 centimorgans. The 1-cM LOD unit support interval spanned 18 cM, from 17 to 35 cM (9,706,961 to 21,871,858 Mb P terminus).

**Table 2:** Loci affecting fibrinogen levels.

Center/Model	Adjusted LOD Score	locus (± 1 LOD interval)	physical locus	Genes* within 1 LOD interval
OK Model 2	2.47	Chr 3 loc 166 cm (157 cm-172 cm)	144,754,119 to 164,001,645	133 Total 30 Labeled

Comparable LOD score from AZ = 0.07 DK = 0.06 ALL = 0.97				

ALL Model 1	2.10	Chr 6 loc 122 cm (113 cm-126 cm)	104,686,675 to 125,240,989	143 Total 30 Labeled

Comparable LOD score from AZ = 1.67 DK = 0.63 OK = 0.83				

DK Model 1	2.81	Chr 7 loc 29 cm (17 cm-36 cm)	9,706,961 to 22,141,263	54 Total 30 Labeled

Comparable LOD score from AZ = 0.02 OK = 0.00 ALL = 0.30				

DK Model 2	3.02	Chr 7 loc 28 cm (17 cm-35 cm)	9,706,961 to 21,871,858	52 Total 30 Labeled

Comparable LOD score from AZ = 0.39 OK = 0.00 ALL = 0.55				

DK Model 2	1.82	Chr 17 loc 76 cm (68 cm-93 cm)	45,179,978 to 66,077,271	240 Total 29 Labeled
Comparable LOD score from AZ = 0.08 OK = 0.00 ALL = 0.42				

* As per the UCSC Genome Browser at

* As per NCBI Map Viewer at .

Further suggestive evidence of linkage to fibrinogen was observed in the Oklahoma center on chromosome 3 at 166 cM. The 1-cM LOD unit support interval spanned 15 centimorgans from 157 – 172 cM (144,754,119 to 164,001,645 Mb). In addition, we found suggestive evidence of linkage to fibrinogen within all centers combined to chromosome 6 at 122 cM. Of the suggestive LOD scores, the only one that declined with adjustment was at chromosome 6, 122 centimorgans, in the analysis including all centers. In a post-hoc analysis, the only available measure of inflammatory state, white blood cell count (WBC), was included with previously described models to adjust for inflammatory state. LOD scores in these analyses showed essentially identical values for OK, model 2, and DK, chromosome 7, model 1 of 2.51 and 2.81 respectively. The LOD score for DK, chromosome 7, model 2 was reduced slightly to 2.41 and became insignificant (0.51) for DK, chromosome 17, model 2.

While the α,β,γ fibrinogen genes are on chromosome 4, the present study shows LOD scores for the DK center only at 4q28, 155cM, ranging between 1.36 for Model 2 to 1.70 for Model 1; none of the other centers exhibited LOD scores over 0.3 in this region.

Assuming a genome-wide heritability of 25%, power calculations indicated an 80% power to detect a locus with a LOD score > 3 for a locus contributing a minimum of between 15 and 20% of total variance for each of the three centers and 10% for all centers combined.

## Discussion

Evidence for a pathophysiologic role of both thrombotic and inflammatory influences on atherosclerosis continues to accumulate. The fact that fibrinogen was shown in 31 prospective studies involving over 150,000 participants (without apparent disease at baseline) to predict future CVD events is compelling, but not absolute, support for a primary role in the development of atherosclerosis. [4] The influence of fibrinogen would appear to be enhanced among those with a high prevalence of diabetes, smoking and other environmental influences [13], including the presently described SHS population [5].

In addition to multiple environmental influences on plasma fibrinogen levels [7,9] the effects of heritability [9,14,15] and specific genetic variants (primarily involving the 3 fibrinogen gene cluster) are well established [19-24,37]

While genetic polymorphisms in the fibrinogen genes are found in diverse populations around the world [38,39], and a role for variants of the α,β,γ fibrinogen genes has been reported [9], there still appears to be substantial variation due to unknown genetic effects [40]. Although the Dakota center found modest genetic effects at the fibrinogen gene cluster on chromosome 4 (LOD 1.7), this was not seen at either of the other two centers. Other investigators have failed to demonstrate linkage to the fibrinogen gene loci in otherwise adequately powered studies [18,41]., It would be of interest to determine if the common polymorphisms (such as the -455G/A variant of the β gene promoter) seen in other populations [22] at this locus are simply not well represented in these centers, or whether they lack the typical effect size.

Suggestive evidence of linkage to novel loci was identified in this study on chromosomes 3 (OK center), 6 (all centers combined) and both 7 and 17 (DK center). Our linkage results at 3q22.2 (166 cM, closest to marker D3S1299) are within 0.5 Mb of the angiotensin II receptor, type 1 gene (*AGTR1*, 149.4 Mb-150.1 Mb) and this gene is thus well within the 1 LOD interval (144.8 Mb-169.8 Mb). The angiotensin converting enzyme I (*ACE1*) gene is also located within the 1 LOD interval of our suggestive locus at chromosome 17 (68 cM to 93 cM) [42] Although *AGTR1*, *ACE1*, and fibrinogen all have relevance to CVD, there is no readily apparent physiologic connection between fibrinogen levels and the renin-angiotensin-system (RAS). While the RAS is an important factor in the regulation of blood pressure, and fibrinogen is often elevated in the presence of hypertension, we are unaware of any evidence that increased fibrinogen preceeds or influences the development of hypertension. It is possible that a genetic variant in this locus simultaneously controlling expression of both fibrinogen and RAS genes.

Findings of Ding et al [41] support our results at 6q21, see Table 3. There are 143 genes within the 1 LOD confidence limits of the locus at 6q21 (122 cM, closest to marker D6S303); but none have obvious relationships with fibrinogen.

**Table 3:** Other studies showing genetic effects on fibrinogen levels.

Variant, and/or locus	Physical locus	LOD score or p-value	Reference
Chromo 1p31.3 rs6683832	62,988,925	5.6 × 10^-5^	Yang et al [41]
1p31 Leptin receptor (LEPR) haplotype block including: rs3790432, rs1137101, rs1805096	65,764,013 – 65,873,699	0.005	Zhang et al [45]
Chromo 2, 55.5 cM (African Americans)	36,013,772 – 36,214,030	LOD = 2.14	Ding et al [41]
Chromo 2, q36.3 rs1273819	227,307,918	LOD = 2.4	Yang et al [41]
Chromo 6, 136 cM (African Americans)	134,596,514 – 134,796,754	LOD = 1.43	Ding et al [41]
Chromo 12, 152.16 cM fibrinogen and CRP (bivariate) (African Americans)	127,228,944 – 127,429,321	LOD = 3.44	Ding et al [41]
Chromo 12q14.2 rs10506540	65,699,364	2.2 × 10^-5^	Yang et al [41]
Chromo 12q24 D12S79 – D12S1718	114,444,811 – 116,237,121	LOD = 2.1	Soria et al [18]
Chromo 14 rs974673	33,550,265	3.1 × 10^-5^	Yang et al [41]
Chromo 14q11 D14S72 – D14S50	20,340,828 – 21,525,933	LOD = 3.12	Soria et al [18]
Chromo 17, 96 cM	67,937,333 – 68,137,655	LOD = 1.4	Yang et al [51]
Chromo 17, 93 cM (African Americans)	66,019,673 – 66,220,040	LOD = 1.95	Ding et al [41]
Chromo 21, 13.05 cM fibrinogen and CRP (bivariate) (Non-Hispanic Whites)	20,322,875–20,523,182	LOD = 3.03	Ding et al [41]

Within the 1 LOD drop interval at 7p21.1 (28 cM, closest to marker D7S507), the integrin beta-8 (*ITGB8*) gene is located at 31 cM. This gene is related to *ITGB3 *(see below) and shares similar important relationships with transforming growth factor, beta 1 (*TGFB1*) and other elements of the immune system [43,44]. The interleukin-6 gene (*IL6*) is located 2 Mb beyond the 1 LOD drop interval on 7p21 at 22.7 Mb, closest to deCODE markers at 41.69 cM. Zhang et al [45] have shown a correlation between *IL6 *and fibrinogen levels (r = 0.22, p < 0.0001). Not all [24], but some *IL6 *polymorphisms are known to affect fibrinogen levels [46]. The central role of *IL6 *in inflammatory physiology would make this a strong candidate for effects on fibrinogen as an acute phase reactant, although the fact that the DK LOD score at this locus was essentially unchanged with adjustment for an inflammatory marker (WBC) suggests inflammatory stimuli are not the sole determinants of fibrinogen levels.

Our findings at chromosome 17, 76 cM (nearest marker D17S1607) are in close proximity to the LOD of 1.4 found by Yang et al [47] at 96 cM (on the Marshfield map). The growth hormone (*GH1*) lies within the 1 LOD drop interval and exerts an influence on fibrinogen levels [48]. The integrin, beta-3 (*ITGB3*) and "signal transducer and activator of transcription 3" (*STAT3*) genes both reside within 3 and 8 Mb respectively of this 17q23.3 interval and both are known to affect baseline and acute phase fibrinogen expression [41,49].

These current findings derive from specific subsets of the North American Indian population and each center of the study analyzes a group essentially the same size as that reported from the Framingham Heart Study [16]. One might expect more homogeneous findings among these American Indian populations; but they are separated by about 1,000 Km and have distinct language and cultural characteristics. The SHS has found similar diversity between centers in the analysis of other phenotypes. The lack of apparent effect in the AZ and OK centers of the fibrinogen gene cluster at chromosome 4q28, in comparison to Caucasian populations, further highlights apparent differences between ethnic groups. No overlapping linkage regions with LOD scores over 1.3 were found in comparing African Americans and non-Hispanic whites in the GENOA study [41], although limitations of power may have been a factor. The relative lack of consistency in linkage study findings has been commented on before and our findings are consistent with this [50]. Perhaps unappreciated environmental effects are present, which have not been fully accounted for in the adjustment strategies used by various investigators.

Strengths of this study include the population-based recruitment with large numbers of participants analyzed and the large, multi-generational families recruited. Our calculations indicate an 80% power to identify a QTL with a LOD score of more than 3 contributing at least 20% of the total phenotypic variance in any center. In spite of the above-mentioned diversity of findings, the genetic background of the populations are likely to be more uniform than population-based studies from urban areas. The prospective collection of a very well characterized phenotype, including many demographic, environmental and physiologic parameters is another strength.

Although the large size of the families in this study increases the power to detect genetic effects, it may also magnify the effects of uncommon polymorphisms in a particular family. This should be minimized by the fact that recruitment for the family study was not based on the presence or history of CVD and the fact that 13.6% of the family study participants were also members of the original, population-based cohort.

## Conclusion

In a minority population with a high prevalence of cardiovascular disease, strong evidence for a novel genetic determinant of fibrinogen levels is found on chromosome 7 at 28 cM. Four other loci, some of which have been suggested by previous studies, were also identified. Fibrinogen is of particular interest since it may act through two independent pathophysiologic mechanisms, i.e. thrombosis and inflammation. A better understanding of the genetic determinants of fibrinogen levels and how they influence the development of atherosclerosis and its complications will assist in fashioning better preventive and treatment strategies for this very serious public health problem.

## Competing interests

The authors declare that they have no competing interests.

## Authors' contributions

All authors have read and approved the final manuscript. LGB: Originated and conducted the majority of the analysis, drafted the manuscript and is the corresponding author. KEN: Provided key oversight and direction of analysis and played a major role in editing the manuscript. XL: Provided initial linear regression analyses, residual files for linkage analysis and editing suggestions. VP: Offered important editorial suggestions for the manuscript. JGU: Supervised laboratory determinations of fibrinogen and made edits to the methods section. JM: Supervised genetic data collection and preparation. SL: Evaluated pedigrees, provided the final pedigree structure and performed the genotypic data cleaning. KH: Provided extensive quality control and data management of the genotyping. HG: Evaluated pedigrees and provided the final pedigree structure VPD: Provided "identical by descent" genotype files for analysis. Evaluated pedigrees and provided the final pedigree structure. LA: Guidance related to statistical linkage analysis. ETL: Phenotypic data collection and quality assurance. RPT: Guidance related to laboratory methodology. SC: Supervised the genotyping and provided important editorial comments.

## Pre-publication history

The pre-publication history for this paper can be accessed here:


